# Serum magnesium and calcium levels in relation to ischemic stroke: Mendelian randomization study

**DOI:** 10.1212/WNL.0000000000007001

**Published:** 2019-02-26

**Authors:** Susanna C. Larsson, Matthew Traylor, Stephen Burgess, Giorgio B. Boncoraglio, Christina Jern, Karl Michaëlsson, Hugh S. Markus, for the MEGASTROKE project of the International Stroke Genetics Consortium

**Affiliations:** From the Unit of Cardiovascular and Nutritional Epidemiology (S.C.L.), Institute of Environmental Medicine, Karolinska Institutet, Stockholm, Sweden; Stroke Research Group, Department of Clinical Neurosciences (M.T., H.S.M.), MRC Biostatistics Unit (S.B.), and Department of Public Health and Primary Care (S.B.), University of Cambridge, UK; Department of Cerebrovascular Diseases (G.B.B.), Fondazione IRCCS–Istituto Neurologico Carlo Besta, Milano, Italy; Department of Clinical Pathology and Genetics (C.J.), Institute of Biomedicine, Sahlgrenska Academy at University of Gothenburg; and Department of Surgical Sciences (K.M.), Uppsala University, Sweden.

## Abstract

**Objective:**

To determine whether serum magnesium and calcium concentrations are causally associated with ischemic stroke or any of its subtypes using the mendelian randomization approach.

**Methods:**

Analyses were conducted using summary statistics data for 13 single-nucleotide polymorphisms robustly associated with serum magnesium (n = 6) or serum calcium (n = 7) concentrations. The corresponding data for ischemic stroke were obtained from the MEGASTROKE consortium (34,217 cases and 404,630 noncases).

**Results:**

In standard mendelian randomization analysis, the odds ratios for each 0.1 mmol/L (about 1 SD) increase in genetically predicted serum magnesium concentrations were 0.78 (95% confidence interval [CI] 0.69–0.89; *p* = 1.3 × 10^−4^) for all ischemic stroke, 0.63 (95% CI 0.50–0.80; *p* = 1.6 × 10^−4^) for cardioembolic stroke, and 0.60 (95% CI 0.44–0.82; *p* = 0.001) for large artery stroke; there was no association with small vessel stroke (odds ratio 0.90, 95% CI 0.67–1.20; *p* = 0.46). Only the association with cardioembolic stroke was robust in sensitivity analyses. There was no association of genetically predicted serum calcium concentrations with all ischemic stroke (per 0.5 mg/dL [about 1 SD] increase in serum calcium: odds ratio 1.03, 95% CI 0.88–1.21) or with any subtype.

**Conclusions:**

This study found that genetically higher serum magnesium concentrations are associated with a reduced risk of cardioembolic stroke but found no significant association of genetically higher serum calcium concentrations with any ischemic stroke subtype.


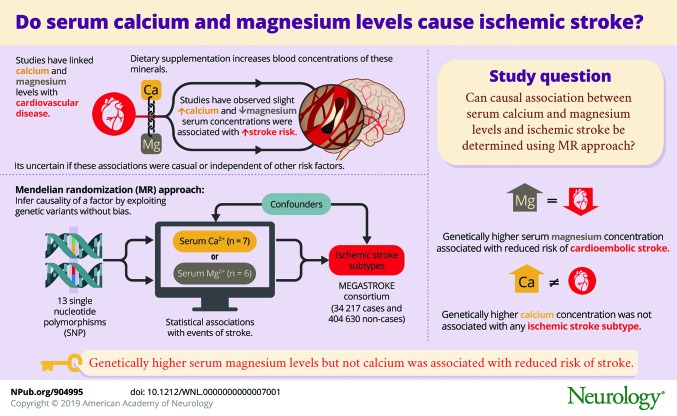


Growing evidence indicates that the essential minerals magnesium and calcium may have a role in cardiovascular disease. Magnesium, the second most predominant intracellular cation, can influence the cardiovascular system through vascular tone, blood pressure, endothelial function, platelet aggregation and coagulation, cardiac arrhythmias, and glucose and insulin metabolism.^1–3^ Calcium, the most abundant mineral in the body, has an essential role in the coagulation system, intracellular signaling, and muscle contraction, but is also associated with some pathologic processes such as carotid artery plaques^4,5^ and calcifications.^6^

Magnesium and calcium supplementation leads to a rise in blood concentrations of these minerals.^7–9^ Therefore, any association of circulating magnesium and calcium concentrations with risk of stroke, an enormous public health problem, can have important public health and clinical implications. Findings from observational epidemiologic studies indicate that low serum magnesium concentrations^10–12^ and slightly elevated serum calcium concentrations^13,14^ are associated with increased risk of stroke. Limited data from randomized controlled trials further indicate that calcium supplementation might increase stroke risk.^4^ However, given the observational design of the majority of available studies on magnesium and calcium in relation to risk of stroke, it is uncertain whether the observed associations are causal and independent of other risk factors, and not biased by reverse causation.

Mendelian randomization (MR) is a genetic epidemiologic method that exploits genetic variants influencing the modifiable exposure of interest as unbiased proxies for the exposure to infer causality.^5,15^ This method has been utilized to demonstrate that serum magnesium^16^ and serum calcium^17^ concentrations are associated with respectively decreased and increased risk of coronary artery disease, but has not been used to determine whether circulating levels of these minerals are associated with risk of ischemic stroke. We applied a 2-sample MR approach to investigate whether serum magnesium and calcium concentrations are causally associated with ischemic stroke as a whole or any of its main subtypes.

## Methods

### Single nucleotide polymorphism selection and data sources

We selected all single nucleotide polymorphisms (SNPs) associated with serum magnesium or calcium concentrations at genome-wide significance (*p* < 5 × 10^−8^) in the largest published genome-wide association studies (GWAS) on these minerals.^18,19^ The GWAS on serum magnesium identified 6 significant and independent (i.e., not in linkage disequilibrium) SNPs, explaining 1.6% of the variance in serum magnesium concentrations, in the joint analysis of the discovery and replication cohorts including 23,829 individuals of European ancestry.^18^ The GWAS on serum calcium identified 7 replicated and independent SNPs, explaining 0.9% of the variance in serum calcium concentrations, in up to 61,079 individuals of European ancestry.^19^

From the MEGASTROKE consortium,^20^ we obtained summary statistics data for stroke for the 13 SNPs. To reduce potential bias caused by population stratification, we restricted the stroke dataset to individuals of European ancestry. Thus, our analyses included data from up to 404,630 noncases and 34,217 ischemic stroke cases, subtyped into cardioembolic stroke (n = 7,193), large artery stroke (n = 4,373), and small vessel stroke (n = 5,386). Stroke subtypes were classified according to the Trial of Org 10172 in Acute Stroke Treatment criteria.^21^

### Standard protocol approvals, registrations, and patient consents

Each study included in the GWAS used in the present study was approved by an institutional review board, and all participants had provided informed consent.

### Statistical analysis

The primary analyses were conducted using the inverse-variance weighted method (hereafter referred to as standard MR analysis), which gives accurate estimates if all SNPs satisfy the instrumental variable assumptions (data available from Open Science Framework, figure e-1, osf.io/b57sq/).^22^ In sensitivity analyses, we used other MR approaches, including the following: (1) the weighted median method, which provides consistent estimates if at least 50% of the weight in the analysis comes from valid instrumental variables^22^; (2) the heterogeneity-penalized model averaging method, which gives consistent estimates if a plurality of the instrumental variables are valid^23^; and (3) the MR-Egger method, which can detect and adjust for pleiotropy.^22,24^ The MR-Egger analysis is disposed to regression dilution bias. The degree of dilution bias was assessed with the *I*^2^_GX_ statistic.^25^ *I*^2^_GX_ values below 0.9 were considered substantial dilution, and the simulation extrapolation (SIMEX) method was used to adjust the estimates for dilution bias.^25^ The MR-PRESSO method was used to detect potential outliers.^26^ Moreover, we conducted sensitivity analyses excluding SNPs with pleiotropic associations with possible confounders or intermediates of the exposure-stroke relationship.

Odds ratios (ORs) were scaled per 0.1 mmol/L (about 1 SD) increase in serum magnesium concentrations and 0.5 mg/dL (about 1 SD) increase in serum calcium concentrations. A Bonferroni-corrected level of significance of less than 0.006 (correcting for 2 exposures and 4 outcomes) was considered statistically significant. Associations of the 13 individual SNPs with the 4 outcomes were considered statistically significant at *p* values of less than 9.6 × 10^−4^. The analyses were conducted using Stata software (StataCorp, College Station, TX) and the MendelianRandomization package^27^ for R. Statistical power was calculated using the method proposed by Brion et al.^28^

### Data availability

All data generated or analyzed during this study are included in the main manuscript and its supplementary information files.

## Results

### Statistical power

We had 100% power to detect an OR of any ischemic stroke of 0.80 for serum magnesium levels and 1.25 for serum calcium levels. The statistical power in analyses of ischemic stroke subtypes is shown in data available from Open Science Framework (table e-1, osf.io/b57sq/).

### Serum magnesium

**Figure 1:**
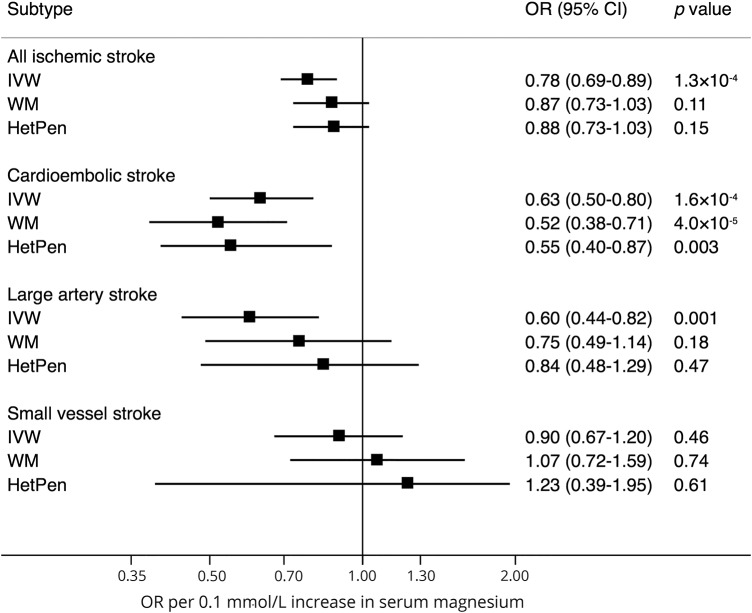
Association between genetically predicted serum magnesium concentrations and ischemic stroke and its subtypes ORs are per genetically predicted 0.1 mmol/L (about 1 SD) increase in serum magnesium concentrations. CI = confidence interval; HetPen = heterogeneity-penalized model-averaging; IVW = inverse variance weighted (standard mendelian randomization method); OR = odds ratio; WM = weighted median.

Of the 6 SNPs associated with serum magnesium concentrations, rs7965584 (near *ATP2B1*) was statistically significantly associated with all ischemic stroke, large artery stroke, and small vessel stroke; rs4072037 (*MUC1*) was associated with cardioembolic stroke; and rs448378 (*MDS1*) was associated with large artery stroke (data available from Open Science Framework, table 2 and figure e-1, osf.io/b57sq/). In the standard MR analysis, genetically predicted serum magnesium concentrations were associated with all ischemic stroke, cardioembolic stroke, and large artery stroke, but not with small vessel stroke (figure 1). The ORs per genetically predicted 0.1 mmol/L (about 1 SD) increase in serum magnesium concentrations were 0.78 (95% confidence interval [CI] 0.69–0.89; *p* = 1.3 × 10^−4^) for all ischemic stroke, 0.63 (95% CI 0.50–0.80; *p* = 1.6 × 10^−4^) for cardioembolic stroke, 0.60 (95% CI 0.44–0.82; *p* = 0.001) for large artery stroke, and 0.90 (95% CI 0.67–1.20; *p* = 0.46) for small vessel stroke (figure 1). Only the association with cardioembolic stroke remained in sensitivity analyses (figure 1).

The *I*^2^_GX_ value from the MR-Egger analysis was 0.87, indicating 13% dilution of the estimates. The MR-Egger analysis, with adjustment for dilution bias using the SIMEX method, provided imprecise estimates (data available from Open Science Framework, table e-3, osf.io/b57sq/). In this analysis, genetically predicted serum magnesium concentrations were associated with cardioembolic stroke but the CI included the null (OR 0.66, 95% CI 0.21–2.10); there was no evidence of directional pleiotropy (data available from Open Science Framework, table e-3). In contrast, directional pleiotropy was detected in the analysis of large artery stroke, and this was not explained by any single SNP (data available from Open Science Framework, table e-3).

The MR-PRESSO analysis identified potential outlying SNPs (at *p* < 0.10), which varied for different subtypes (data available from Open Science Framework, table e-4, osf.io/b57sq/). The association of genetically predicted serum magnesium concentration with cardioembolic stroke persisted after exclusion of the outlier in *TRPM6* (OR 0.56, 95% CI 0.43–0.73). The association also remained after exclusion of 2 SNPs associated with estimated glomerular filtration rate and 1 SNP associated with blood pressure and serum urate levels, but was attenuated after omitting 2 SNPs associated with atrial fibrillation (OR 0.73, 95% CI 0.52–1.03) (data available from Open Science Framework, table e-5).

### Serum calcium

**Figure 2:**
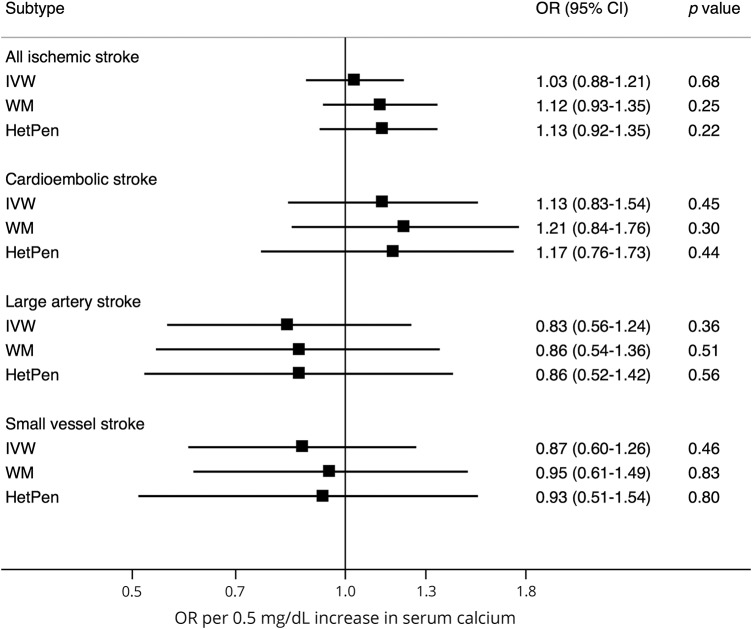
Association between genetically predicted serum calcium concentrations and ischemic stroke and its subtypes ORs are per genetically predicted 0.5 mg/dL (about 1 SD) increase in serum calcium concentrations. CI = confidence interval; HetPen = heterogeneity-penalized model-averaging; IVW = inverse variance weighted (standard mendelian randomization method); OR = odds ratio; WM = weighted median.

None of the calcium-associated SNPs was statistically significantly associated with ischemic stroke as a whole or any subtype (data available from Open Science Framework, table e-6 and figure e-2, osf.io/b57sq/). There were no associations between genetically predicted serum calcium concentrations and any stroke outcome in the standard MR analysis (figure 2). The OR of all ischemic stroke per genetically predicted 0.5 mg/dL (about 1 SD) increase in serum calcium concentrations was 1.03 (95% CI 0.88–1.21; *p* = 0.68). The lack of association remained in sensitivity analyses (figure 2), and there was no evidence of directional pleiotropy in the MR-Egger analysis (data available from Open Science Framework, table e-7, osf.io/b57sq/). The *I*^2^_GX_ value was 0.96, indicating no significant dilution bias in the MR-Egger analysis. No associations of genetically predicted serum calcium concentrations with any stroke outcome were observed after exclusion of the SNP in *GCKR*, which has pleiotropic associations with potential confounders (e.g., blood lipids and type 2 diabetes) (data available from Open Science Framework, table e-8).^17^ No outliers were identified in the MR-PRESSO analysis.

## Discussion

Findings of this MR study showed a consistent association between genetically higher serum magnesium concentrations and reduced risk of cardioembolic stroke but not other subtypes. Genetically predicted serum calcium concentrations were not associated with any ischemic stroke subtype or with ischemic stroke overall.

Although several observational prospective studies have reported that low circulating magnesium concentrations^10–12^ and low magnesium intake^29^ are associated with increased risk of stroke, data on ischemic stroke subtypes are scarce.^12^ In the Nurses' Health Study, low plasma magnesium concentrations (<0.82 mmol/L) were associated with an approximately 70% to 80% increased risk of embolic and thrombotic stroke,^12^ supporting our findings. Previous observational studies were limited by possible residual confounding because low magnesium concentrations and magnesium intake are correlated with potential risk factors for stroke.^10–12^

Magnesium may in part reduce the risk of cardioembolic stroke through its antiarrhythmic effects^1,3^ and via atrial fibrillation. Low serum magnesium concentrations are associated with increased risk of atrial fibrillation,^30,31^ which is a strong risk factor for cardioembolic stroke. Two of the magnesium-associated SNPs were significantly associated with atrial fibrillation, including the SNPs in the *MUC1* (*p* = 0.02) and *SHROOM3* (*p* = 2.4 × 10^−4^) genes, with the allele associated with higher serum magnesium concentrations being associated with lower risk of atrial fibrillation.^32^ The association between genetically predicted serum magnesium concentrations and cardioembolic stroke was attenuated after exclusion of those 2 SNPs, suggesting that the association may partly be mediated by atrial fibrillation.

Magnesium also has anticoagulant and antiplatelet properties.^1,3^ Magnesium is considered to be nature's calcium blocker as it suppresses many of the physiologic actions of calcium.^1,3^ For example, calcium promotes blood coagulation, whereas magnesium suppresses blood clotting and thrombus formation and reduces platelet aggregation, the synthesis of platelet agonist thromboxane A2, von Willebrand factor binding to collagen, and thrombin-stimulated calcium influx.^1,3,33–35^ Antithrombotic effects may lead to reduction in risk of both cardioembolic and large artery stroke. A significant association between genetically predicted serum magnesium concentrations and large artery stroke was observed in the standard MR analysis, but this association did not persist in sensitivity analyses.

Other possible mechanisms whereby high serum magnesium concentrations may reduce ischemic stroke risk include improvement of endothelial function^36,37^ and reduction in blood pressure,^36,38^ atherosclerotic calcification,^39^ arterial stiffness,^40^ oxidative stress,^41^ fasting glucose concentration,^38^ insulin resistance,^42^ and risk of type 2 diabetes.^43,44^ Some of those beneficial effects may also lead to a reduction in small vessel stroke, which was not observed in this study.

The MR design has not been previously used to determine the association between serum calcium concentration and risk of ischemic stroke, but a few observational prospective studies have examined the association between serum calcium concentrations and risk of stroke.^13,14^ In a cohort of about 440,000 Swedish adults, high (≥2.40 mmol/L) vs low (<2.25 mmol/L) serum calcium concentrations were associated with a 12% increased risk of incident ischemic stroke and with a 40% increased risk of fatal ischemic stroke.^14^ Another cohort of 13,288 US adults showed a 16% increase in risk of total stroke per 1-SD increase in serum calcium concentrations.^13^ The association of genetically predicted serum calcium concentration with cardioembolic stroke in the present study was of similar magnitude, though nonsignificant, as the association with stroke in previous observational studies^13,14^ and with coronary artery disease in a previous MR study (OR 1.25, 95% CI 1.08–1.45).^17^ The estimates for serum calcium and cardiometabolic stroke are also similar to those for calcium supplementation and stroke from a meta-analysis of 8 randomized controlled trials (relative risk 1.15, 95% CI 1.00–1.32; *p* = 0.06).^4^ It is unclear why genetically predicted serum calcium concentrations were not associated with large artery stroke, which like coronary heart disease is related to atherosclerosis. A possibility is that we may have overlooked an association because of low power (data available from Open Science Framework, table e-1, osf.io/b57sq/).

A major strength of this MR study is that biases that can be of concern in conventional observational studies were avoided. Other important strengths are the large number of cases of ischemic stroke and that associations with ischemic stroke subtypes could be investigated.

A limitation is that statistical power was low in the analyses of ischemic stroke subtypes. The power was particularly low in the analyses of calcium because the SNPs only explained a small proportion of the variance (0.9%) in serum calcium levels. Hence, we cannot rule out that we may have overlooked weak associations between genetically predicted serum calcium concentrations and ischemic stroke subtypes. Another shortcoming is that the biological function of several of the genetic loci associated with serum magnesium and calcium levels are unknown (data available from Open Science Framework, table e-9, osf.io/b57sq/).

The reliability of MR results relies on 3 main assumptions (data available from Open Science Framework, figure e-1, osf.io/b57sq/), which can be violated by population stratification, canalization, and pleiotropy. Population stratification was minimized because we restricted the study populations to European-descent individuals. We could not directly test whether canalization may have influenced the results. Canalization refers to compensatory processes during development that alleviate the genetic effect. Such feedback mechanisms would bias the results toward the null and cannot explain the observed association between serum magnesium concentration and cardioembolic stroke. Pleiotropy occurs when a genetic variant is associated with more than one phenotype. We conducted several sensitivity analyses to explore and adjust for pleiotropy. The association of genetically predicted serum magnesium concentrations with cardioembolic stroke, but not the other subtypes or overall stroke, was robust in these sensitivity analyses and the MR-Egger analysis provided no evidence of directional pleiotropy.

This study found evidence that genetically higher serum magnesium concentrations may be associated with a reduced risk of cardioembolic stroke. Genetically higher serum calcium concentrations were not associated with ischemic stroke, but the existence of an effect of low magnitude cannot be ruled out.

## Glossary

CI: confidence interval
GWAS: genome-wide association study
MR: mendelian randomization
OR: odds ratio
SIMEX: simulation extrapolation
SNP: single nucleotide polymorphism
